# Fascia-Focused Versus Conventional Physiotherapy for Chronic Low Back Pain and Comorbid Depression in Psychosomatic Inpatients

**DOI:** 10.3390/jcm15103698

**Published:** 2026-05-11

**Authors:** Lea Overmann, Katharina Steinmeier, Andreas Brandl, Christoph Egner, Andrea Kreutzer, Sonia Gadea de Reckel, Petrilena-Sorina Floroiu, Silke Zimmermann, Daniel Stühn, Heike Geiß, Annette Kleeberg, Robert Schleip

**Affiliations:** 1 Private Clinic Overmann, 59229 Ahlen, Germany; overmann.lea@gmx.de; 2 Association for Fascia Research, 80799 Munich, Germany; 3 Department of Health and Psychology, DIPLOMA University of Applied Sciences, 37242 Bad Sooden-Allendorf, Germany; katharinasteinmeier@gmx.de (K.S.); andreas-rudi.brandl@tum.de (A.B.); christoph.egner@diploma.de (C.E.); 4 Department of Conservative and Rehabilitative Orthopedics, TUM School of Medicine and Health, Technical University of Munich, 80809 Munich, Germany; 5 Department of Orthopedics and Psychosomatic Medicine, Klinik Kurhessen, 37242 Bad Sooden-Allendorf, Germany; bowtech-bsa@gmx.de (A.K.); sonia.gadea-de-reckel@drv-hessen.de (S.G.d.R.); sorina.floroiu@yahoo.com (P.-S.F.); silke.zimmermann@drv-hessen.de (S.Z.); daniel.stuehn@drv-hessen.de (D.S.); geiss.heike68@gmail.com (H.G.); annette@familie-kleeberg.de (A.K.); 6 Triagon Academy, MRS 1331 Marsa, Malta

**Keywords:** Bowen therapy, fascia training, psychosomatic rehabilitation, multimodal treatment, pain rehabilitation, depressive symptoms

## Abstract

**Background:** Chronic low back pain (CLBP) and depression often co-occur. This study compared a fascia-focused physiotherapeutic program with a conventional physiotherapeutic and relaxation-based program in psychosomatic inpatients with CLBP and comorbid depression. **Methods:** In this exploratory quasi-randomized study, 41 inpatients were allocated to a fascia-focused intervention group (*n* = 23) or a conventional active control group (*n* = 18). Over six weeks, the intervention group received Bowen therapy and fascia circuit training, whereas the control group received progressive muscle relaxation and strength circuit training. Outcomes were assessed at baseline and after rehabilitation. NRS pain intensity and BDI-II depressive symptom severity were the main clinically relevant outcomes; spinal function, tissue stiffness, pressure pain threshold, and craniovertebral angle were secondary or exploratory outcomes. **Results:** Both groups improved over time in pain intensity and depressive symptom severity. NRS scores decreased by 3.21 ± 2.61 points in the fascia-focused group and by 2.17 ± 2.34 points in the control group; BDI-II scores decreased by 9.30 ± 13.42 and 7.22 ± 8.57 points, respectively. Repeated-measures ANOVA confirmed significant time effects for NRS and BDI-II, with no significant group differences or time × group interactions. Significant time effects were also observed for thoracic tissue stiffness, lumbar and pelvic posture, thoracic and lumbar mobility, and pelvic stability. **Conclusions:** Fascia-focused and conventional physiotherapy showed similar observed effects in this exploratory quasi-randomized study. The absence of significant between-group differences should be interpreted cautiously because the study was not designed or powered to establish formal equivalence.

## 1. Introduction

Chronic low back pain (CLBP) is one of the leading causes of disability worldwide and is associated with substantial personal, social, and economic burden [1,2,3]. Clinical studies have consistently shown a high degree of comorbidity between chronic pain and depression. Their co-occurrence can exacerbate both disorders, leading to poorer physical functioning, diminished quality of life, and reduced treatment responsiveness [1,4,5]. For patients with CLBP and comorbid depressive symptoms, rehabilitation therefore needs to address not only pain intensity and physical function, but also psychosocial and affective factors that may influence pain perception, movement behavior, and treatment response.

Current recommendations highlight a biopsychosocial, mainly non-surgical approach to CLBP management. Depending on the clinical presentation, common components include education and advice, exercise-based interventions, selected manual therapies, psychological or cognitive–behavioral strategies, and multicomponent rehabilitation [6]. For patients with persistent pain and significant psychosocial burden, multimodal rehabilitation is especially relevant because it targets both physical and psychological factors contributing to chronic pain [6,7]. In inpatient psychosomatic rehabilitation, however, patients usually receive structured multimodal care rather than isolated single-component interventions. Therefore, the clinically relevant question is not only whether a specific treatment approach can improve symptoms, but whether it provides added value compared with another active and feasible rehabilitation package.

Patients with CLBP and comorbid depression in psychosomatic inpatient rehabilitation may differ from general CLBP populations because pain-related, affective, perceptual, and behavioral factors are often closely intertwined. Depression is associated with poorer health outcomes in patients with low back pain and may be accompanied by altered pain processing, increased pain sensitivity, reduced activity, fear-avoidance behavior, and protective movement patterns [1,4,5]. In addition, emotional distress and disturbed body perception or interoceptive processing may contribute to pain-related disability and reduced responsiveness to unimodal treatment approaches [8]. Therefore, body-oriented and movement-based interventions may be particularly relevant in psychosomatic rehabilitation, where treatment often aims to address physical function, pain experience, emotional regulation, and body awareness within an integrated multimodal framework.

Within this context, fascia-focused interventions have gained increasing attention. These include manual techniques targeting myofascial structures and movement-based methods aimed at enhancing tissue mobility, load transfer, body awareness, and sensorimotor regulation [8,9]. Their potential relevance for CLBP management lies in their compatibility with multimodal care and in their possible effects on pain, movement quality, and body awareness. However, since fascia-focused interventions are often delivered as part of complex treatment approaches, further comparative research is needed to clarify whether their effects exceed those of other active rehabilitation strategies [10,11].

Interest in fascia is partly based on the growing recognition of fascia as a body-wide connective tissue network that is mechanically relevant and richly innervated. Fascial tissues have been linked to nociception, proprioception, interoception, and autonomic regulation, which provides a theoretical rationale for considering fascia in chronic pain rehabilitation [8,12]. These mechanisms may be particularly relevant in psychosomatic inpatients with CLBP and comorbid depression, because chronic pain, affective symptoms, altered movement behavior, and disturbed body perception may interact in this population. However, biological plausibility does not establish clinical superiority. It remains unclear whether fascia-focused interventions provide clinically meaningful advantages over established active rehabilitation strategies [12].

Among fascia-oriented manual approaches, the Bowen technique is notable for combining gentle rolling movements with pauses intended to promote self-regulation and relaxation [13]. Recent fascia-focused clinical studies on musculoskeletal pain have reported promising yet mixed findings, although evidence of superiority over other active rehabilitation strategies remains limited. Isolated manual or fascia-directed interventions have been associated with short-term reductions in fascial stiffness and improvements in pain-related outcomes immediately after treatment [12,14,15]. Rehabilitation-focused fascia approaches have also shown benefits in mobility, movement-related pain, and quality of life over a six-week treatment period [11]. However, the available evidence does not yet sufficiently clarify whether fascia-focused approaches are superior to other active rehabilitation strategies, particularly in complex psychosomatic inpatient populations with both CLBP and comorbid depression.

This comparative gap is clinically and practically important. Fascia-focused programs may require specific therapist expertise, manual-treatment competence, and structured exercise instruction, whereas conventional programs such as progressive muscle relaxation and strength-oriented circuit training are already widely established in rehabilitation settings. Therefore, the implementation of fascia-focused approaches in inpatient psychosomatic rehabilitation requires evidence on whether they provide measurable added value over conventional rehabilitation packages. Such comparative evidence is also relevant to clinical decision-making because treatment selection in rehabilitation settings must consider not only clinical outcomes but also feasibility, therapist training requirements, treatment time, and the efficient use of available resources.

The present study compared two active multimodal rehabilitation packages in psychosomatic inpatients with CLBP and comorbid depression. The fascia-focused package combined Bowen therapy with fascia circuit training, whereas the conventional package combined progressive muscle relaxation with strength circuit training. The study aimed to compare their effects on pain intensity, depressive symptom severity, and postural and functional spinal outcomes. In addition, physical measures related to spinal function and myofascial tissue properties, such as tissue stiffness, were included to explore whether outcomes theoretically linked to myofascial function differed between the two approaches. We hypothesized that both interventions would be associated with improvements after six weeks of inpatient rehabilitation, but that the fascia-focused program might show greater effects on outcomes more closely related to myofascial function, particularly tissue stiffness, posture, and mobility.

## 2. Methods

### 2.1. Study Design and Setting

This study was a quasi-randomized clinical trial conducted in an inpatient psychosomatic rehabilitation setting. The research took place at Klinik Kurhessen, Haus Werra, within the Department of Psychosomatic Medicine at DRV Hessen. The intervention period lasted six weeks, consistent with the typical duration of inpatient psychosomatic rehabilitation. The study was approved by the Ethics Committee of DIPLOMA University of Applied Sciences in December 2024 (reference number 1176/2024) and was conducted in accordance with the principles of the Declaration of Helsinki. The study was registered with the German Clinical Trials Register on 10 April 2025 (DRKS00036589), prior to the start of recruitment on 11 April 2025. Recruitment and data collection were completed on 30 May 2025. Written informed consent was obtained from all participants prior to inclusion.

### 2.2. Participants

Participants were consecutively recruited from incoming patients admitted to the inpatient psychosomatic rehabilitation program at Klinik Kurhessen. Eligible patients had clinically documented depression and CLBP as part of the routine clinical admission process. The four responsible physicians screened potential participants against the predefined inclusion and exclusion criteria and informed eligible patients about the study procedures. Inclusion criteria were active participation in the inpatient rehabilitation program, the presence of CLBP, and comorbid depression. Exclusion criteria involved an inability to independently transfer from a lying to a standing position, which would have prevented participation in the circuit training. Detailed clinical characteristics such as pain duration, pain subtype, pain distribution, medication use, relevant somatic or psychiatric comorbidities, and prior treatment history were not systematically documented in the study dataset.

### 2.3. Allocation and Blinding

After providing written informed consent, participants were allocated to the study groups in the order of enrollment. This pragmatic allocation procedure was feasible in the inpatient rehabilitation setting, but it did not constitute true randomization because neither a computer-generated random sequence nor allocation concealment was used [16]. Consequently, the study should be considered quasi-randomized, and potential selection bias or baseline imbalance cannot be ruled out.

No stratification or matching by sex, symptom severity, medication use, pain characteristics, or other potential confounders was performed. Group comparability was assessed after allocation using the available baseline demographic and clinical outcome variables. Group allocation was performed by an external researcher who was not involved in treatment delivery or outcome assessments. However, because the allocation sequence was predictable in principle, this procedure offered less methodological control than a fully randomized controlled trial.

Participants were not explicitly informed which intervention group they had been assigned to and had no direct exposure to the other group’s procedures; they were therefore considered blinded with respect to treatment allocation. However, therapists and the outcome assessor could not be blinded because of the nature of the interventions and the clinical setting.

### 2.4. Interventions

The study compared two active multimodal rehabilitation packages rather than isolated treatment components. The fascia-focused rehabilitation package consisted of Bowen therapy combined with fascia circuit training, whereas the conventional active rehabilitation package consisted of progressive muscle relaxation combined with strength circuit training. Therefore, the design allows comparison of these two clinically applicable treatment packages, but does not allow the effects of individual components to be isolated. Both circuit-training programs followed the same practical structure and dose. Each session lasted approximately 30 min and included a brief common warm-up followed by six exercise stations. Each station lasted 1 min, followed by a 1 min transition period. Depending on the available session time and individual pace, participants typically completed approximately 1.5 to 2 rounds per session. Exact session-level data on the number of completed rounds were not systematically recorded.

### 2.5. Bowen Therapy and Fascia Circuit Training

Participants in the intervention group received five 45 min Bowen therapy sessions (roughly one session per week) and ten 30 min fascia circuit-training sessions (about two sessions per week) over six weeks, depending on each individual’s rehabilitation schedule. Bowen therapy is a gentle manual fascial technique involving specific rolling movements performed over muscles, tendons, and fascia with the fingers or thumbs, applied transversely to muscle fibers. Each movement offers a brief, low-intensity mechanical stimulus, followed by a short pause of approximately two minutes to allow for neuromyofascial integration. This process aims to promote the reorganization of neuromuscular and fascial tension patterns through afferent stimulation and autonomic control. In participants with CLBP, treatment focused on the lumbosacral fascia, erector spinae, gluteal muscles, and the posterior thigh, with additional movements at the cervicothoracic junction to address fascial continuity and postural regulation [17]. Each session included about 10–14 standardized movements. Treatments were conducted in small groups of up to three participants. The intensity of manual stimulation was low and adjusted to the participant’s comfort. The fascia circuit training was performed in groups and comprised six predefined exercise stations. If the full circuit could not be completed within the session time, participants were instructed to continue at the next session at the station where they had left off. For each station, three exercise options with graded difficulty levels (easy, medium, and hard) were provided using station cards, primarily with visual instructions. Additionally, a flipchart with exercise descriptions was available in the room. Sessions were supervised by a physiotherapist. The exercises were developed by expert groups, including physiotherapists from the clinic and fascia researchers, and were designed to target myofascial chains relevant to lumbar stability and functional movement. Unlike the control condition, this intervention aimed to combine fascia-oriented manual treatment with movement-based exercises emphasizing elastic loading, myofascial chain activation, and whole-body movement integration, rather than conventional muscular strengthening or a standardized verbal relaxation protocol.

### 2.6. Control Group: Progressive Muscle Relaxation and Strength Circuit Training

The control condition was designed to represent an established non-fascial physiotherapeutic and relaxation-based rehabilitation approach within the inpatient psychosomatic setting. This allowed comparison of the fascia-focused program against a clinically relevant conventional treatment rather than against no treatment.

Participants in the control group received five 45 min progressive muscle relaxation (PMR) sessions (approximately one session per week) and ten 30 min strength circuit-training sessions (approximately two sessions per week) over a period of six weeks, depending on the individual rehabilitation schedule. PMR sessions followed Edmund Jacobson’s standardized protocol and were conducted in groups of approximately 12 participants in a fitness room under the therapist’s guidance [18]. Participants were instructed to tense and subsequently relax predefined muscle groups in a fixed sequence, focusing on the perception of tension and subsequent relaxation. After completing the sequence, participants rested for a short period while listening to relaxing music before the session ended. The intensity of PMR was standardized through verbal guidance from the therapist and focused on alternating voluntary muscle tension and relaxation, with no physical exertion beyond the instructed contractions.

The strength circuit training mirrored the fascia circuit in overall structure and scheduling. It consisted of six predefined stations; each performed for 1 min with a 1 min transition period between stations. If a participant did not complete the full circuit, the next session resumed at the last completed station. At each station, three graded exercise options (easy, medium, and difficult) were provided using station cards, and a flipchart with exercise descriptions was available in the room. Sessions were supervised by a physiotherapist and included visual instructions and guidance throughout. A detailed overview of the exercises included in the strength and fascia circuit programs is provided in Supplementary Table S1 to improve reproducibility.

### 2.7. Outcome Measures

The selected outcome measures were chosen to capture complementary domains relevant to the study objective, including subjective pain intensity, mechanical pain sensitivity, myofascial tissue properties, and spinal posture and function. Baseline measurements were taken upon admission before the start of the assigned intervention, and follow-up measurements were performed after completion of the 6-week rehabilitation program.

Given the exploratory character of the study, no single confirmatory primary endpoint was predefined. However, pain intensity, assessed using the NRS, was considered the main clinically relevant outcome. Depressive symptom severity, assessed using the BDI-II, was considered a secondary clinically relevant outcome. Tissue stiffness, PPT, CVA, and spinal posture, mobility, and stability were treated as secondary or exploratory physical outcomes. Additional patient-reported variables were collected to characterize clinically relevant psychological and treatment-related aspects of the sample, including treatment perception and acceptance, treatment credibility/expectancy, and pain-related behavior. These variables were not treated as primary outcomes.

### 2.8. Main Clinical Outcome

NRS: Pain perception was assessed using an NRS, a straight 10-point line with endpoints labeled “no pain” (1) and “pain as bad as it could be” (10). Participants indicated their perceived pain intensity on the scale. The NRS is a widely recognized instrument for evaluating subjective pain and has demonstrated validity for detecting both increases and decreases in pain [19].

### 2.9. Secondary Clinically Relevant Outcome

Beck Depression Inventory-II (BDI-II): A 21-item self-report instrument to determine the severity of depressive symptoms across affective, cognitive, motivational, behavioral, and somatic domains. Scores were interpreted as minimal (0–13), mild (14–19), moderate (20–28), or severe (29–63) [20,21]. The BDI-II was used to quantify the severity of depressive symptoms and not to establish the clinical diagnosis of depression.

### 2.10. Exploratory Physical Outcomes

IdentoPRO: Myofascial tissue stiffness and PPT were evaluated using the IdentoPRO, a digital indentometry device designed to measure tissue stiffness, elasticity, and mechanical pain sensitivity. Previous studies have shown high intra- and inter-rater reliability and good accuracy for this instrument, and its validity has also been tested in a phantom model study [22,23]. Measurements were taken with participants lying prone to enable precise identification of anatomical landmarks. Stiffness was recorded at three specific sites: 2 cm to the right of Th7, 2 cm to the right of L2/3, and at the thenar eminence. For the paraspinal regions (Th7 and L2/3), an indentation depth of 8 mm was used, while 5 mm was used at the thenar site. The probe was pressed straight onto the tissue until an acoustic signal indicated that the desired indentation depth had been achieved, after which stiffness values (N/mm) were recorded, with lower values indicating less tissue stiffness [24]. PPT was measured by gradually increasing pressure with the probe while participants verbally indicated the first sensation of pain, defined as the shift from pressure to a burning, stabbing, drilling, or pulling sensation [24]. All measurements were performed three times, and the average was used for analysis. For stiffness measurements, the device’s variation coefficient had to remain below 15% to ensure acceptable within-session consistency.

TricuroGO: Spinal posture, mobility, and stability were evaluated using the TricuroGO, a digital back-assessment device operated via a mobile app [25]. The system provides region-specific metrics for the thoracic spine, lumbar spine, and pelvis. In the present study, the TricuroGO sensor was attached to the smartphone on the left side and connected to the corresponding mobile application before testing. Measurements were performed with participants standing in front of the examiner with the upper body uncovered, or partially uncovered when appropriate, to allow unobstructed assessment along the spine. Each assessment was repeated three times under standardized conditions. For all measurements, recording began at the level of C7 and ended at the midpoint of the sacrum, while the device was guided along the spine, maintaining contact between the two guide wheels and the body surface. Three standardized test positions were used: (1) upright standing with feet hip-width apart and arms hanging loosely to assess posture, (2) forward flexion of the spine to assess mobility, and (3) a modified Matthiass test to assess postural stability [26]. During the stability test, participants held a total weight of 822 g with both arms extended forward for 30 s. After completion of the three measurements, the application generated summary scores for posture, mobility, and stability, including separate values for the thoracic, lumbar, and pelvic regions. The interpretation of the recorded values followed the manufacturer’s guidance documents.

Angle Meter: Forward head posture was assessed using an angle meter to measure the CVA. Participants stood upright with their feet positioned on a marked line to ensure consistent stance alignment. Lateral profile photographs were taken using an iPhone 12 Pro Max (Apple Inc., Cupertino, CA, USA) running iOS 18.3.1, mounted on a fixed tripod. Before image acquisition, two anatomical landmarks—the vertebra prominens (C7) and the external auditory meatus—were identified and marked. Angle measurements were obtained using the smartphone inclinometer application Angle Meter (iOS platform, version 1.8.3; A. Kozlov). The CVA was first estimated using the smartphone’s built-in image editor and then verified with the Angle Meter application’s measurement function. All technical and procedural specifications for this assessment were applied as described by [27].A CVA of <50° was interpreted as indicating forward head posture, whereas values ≥ 55° were considered within the normal range [27].

**Figure 1:**
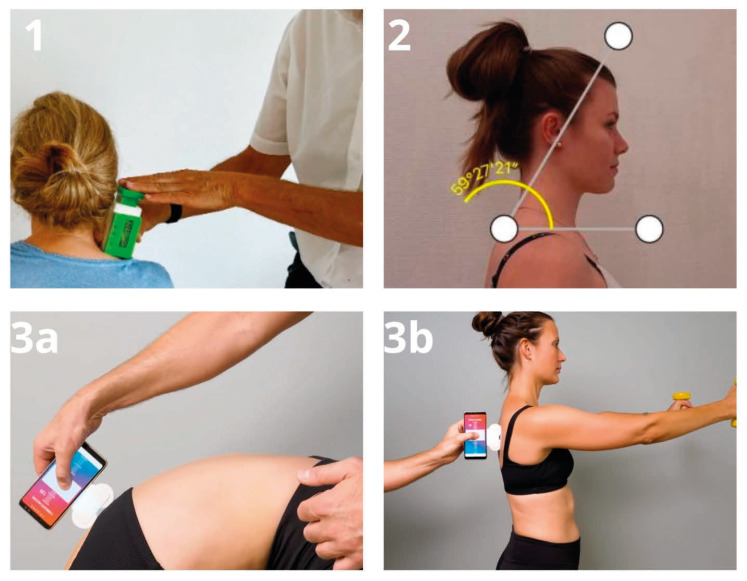
Measurement procedures and devices used for the assessment of tissue stiffness, pressure pain threshold, posture, mobility, and postural stability. (**1**) Application of the IdentoPRO device for assessing myofascial tissue stiffness and pressure pain threshold (PPT). The probe was applied perpendicularly to the tissue surface until the predefined indentation depth was reached. (**2**) Assessment of the craniovertebral angle (CVA) using the smartphone-based Angle Meter application. Standardized lateral photographs were obtained with the vertebra prominens and external auditory meatus marked as anatomical landmarks. (**3a**) Placement of the TricuroGO sensor on the lumbar region during forward flexion to assess spinal mobility. Motion data were recorded digitally via the corresponding mobile application. (**3b**) Placement of the TricuroGO sensor on the thoracic region during a modified Matthiass test to assess thoracic postural stability. Participants held a lightweight object with both arms extended forward during the measurement.

Photographs illustrating the application and handling of the measurement instruments are provided in Figure 1.

In addition, patient-reported outcomes were collected to capture participants’ mood, pain-related behavior, and perceptions of the therapy, which are considered relevant factors in CLBP and may influence myofascial properties [28]. These included treatment perception and acceptance, treatment credibility/expectancy, and pain-related behavior. These variables were assessed using the questionnaires described below.

### 2.11. Patient-Reported Variables

Helping Alliance Questionnaire (HAQ): Administered after explanation of the therapies to assess participants’ subjective perception and acceptance of the treatment. Responses were scored on a 1–9 scale, with one indicating “not at all applicable” and nine indicating “very applicable” [29].

Credibility/Expectancy Questionnaire (CEQ): A modified excerpt was administered after completion of the therapies to evaluate participants’ perception of the therapeutic relationship. The German version of the CEQ is a reliable tool, characterized by high internal consistency and test–retest reliability. Participants respond to six items using a 9-point numeric rating scale [30].

Pain Behavior Questionnaire (FSV): Used to quantify pain-related behaviors across four domains: avoidance, cognitive control, social support, and activity. T-scores were calculated based on reference data from patients with rheumatic diseases, with reliability ranging from α = 0.68 to 0.84 across scales [31].

### 2.12. Statistical Analysis

Baseline comparability between intervention and control groups was assessed using independent-samples t-tests or Mann–Whitney U tests, depending on the distributional characteristics of the data. Continuous variables are presented as mean ± standard deviation (SD) for approximately normally distributed data or as median where appropriate. Categorical variables are reported as absolute frequencies and percentages.

Outcomes were analyzed using repeated-measures ANOVA (RM-ANOVA) in a two-group pre–post design to examine the main effects of time and group, as well as the time × group interaction within a single model. RM-ANOVA was employed to maintain a consistent analytical approach across outcomes and to directly test the interaction between time and group. Estimated marginal means were calculated using the emmeans package in R (version 4.1; afex package).

For the main clinical outcomes, NRS pain intensity and BDI-II depressive symptom severity, additional descriptive change analyses were performed to improve clinical interpretability. Absolute change was calculated as the baseline value minus the post-treatment value, so that positive values indicated improvement. Mean changes and between-group differences in change are reported with 95% confidence intervals. Within-group standardized change was expressed as Cohen’s dz, calculated as the mean individual change divided by the standard deviation of the individual change scores. Between-group effect sizes for change were calculated as Cohen’s d, based on the difference in mean change scores divided by the pooled standard deviation of the change scores.

All analyses were performed in Jamovi (version 2.3) and R, using an MRAN snapshot from 1 January 2022, to ensure reproducibility. Statistical significance was set at *p* < 0.05.

No a priori sample size calculation was performed because this study was conducted within the practical constraints of an inpatient rehabilitation setting and should therefore be interpreted as exploratory. Clinically meaningful change was not assessed using predefined minimal clinically important difference thresholds, reliable change indices, or responder analyses. Clinical relevance was therefore interpreted descriptively and cautiously based on the direction of change, statistical significance, and effect size.

## 3. Results

### 3.1. Participant Flow and Baseline Characteristics

**Figure 2:**
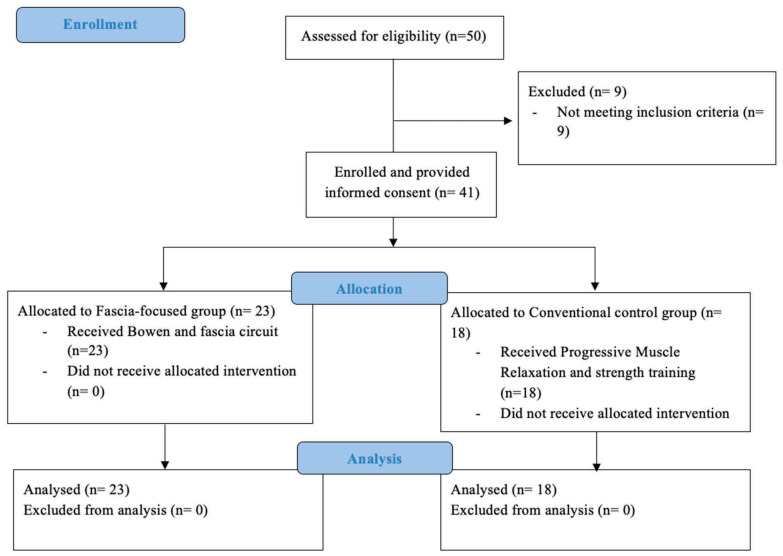
Study flow diagram of participant recruitment, allocation, and analysis. The diagram shows the number of individuals assessed for eligibility, excluded before enrollment, allocated to the fascia-focused intervention group or the conventional active control group, and included in the final analysis. Because the study used alternating allocation rather than full randomization, the diagram is presented as a CONSORT-informed study flow diagram.

A total of 50 individuals were assessed for eligibility; 41 met the inclusion criteria, provided informed consent, and were enrolled in the study. These 41 participants were allocated to the fascia-focused rehabilitation group (*n* = 23) or the conventional active rehabilitation group (*n* = 18), and all allocated participants were included in the final analysis. A detailed CONSORT-informed study flow diagram is provided in Figure 2.

No significant differences were observed between groups in age, sex distribution, pain intensity, depressive symptoms, or other demographic characteristics at baseline, indicating that the study population was comparable in these respects. Effect sizes, calculated using biserial rank correlations, ranged from 0.0121 to 0.1425, corresponding to very small to minor effects. This suggests that the distributions of these characteristics were similar across groups and that these variables are unlikely to confound subsequent analyses. Baseline characteristics of participants in each group are presented in Table 1.

Pain intensity and depressive symptom severity decreased in both groups over the rehabilitation period. Descriptive pre- and post-treatment values, absolute changes, 95% confidence intervals, and standardized effect sizes for NRS and BDI-II are presented in Table 2.

For NRS pain intensity, the fascia-focused intervention group showed a decrease from 6.99 ± 1.90 at baseline to 3.78 ± 2.58 after treatment, corresponding to a mean reduction of 3.21 ± 2.61 points (95% CI 2.08 to 4.34). In the conventional active control group, NRS scores decreased from 6.49 ± 1.92 to 4.32 ± 3.09, corresponding to a mean reduction of 2.17 ± 2.34 points (95% CI 1.01 to 3.33). The between-group difference in reduction was 1.04 NRS points (95% CI −0.53 to 2.60), descriptively favoring the fascia-focused group, although the confidence interval included zero. Within-group effect sizes were large in both groups (Cohen’s dz = 1.23 for the fascia-focused group and dz = 0.93 for the control group). The between-group effect size for change was small to moderate (Cohen’s d = 0.42). Repeated-measures ANOVA confirmed a significant main effect of time for NRS scores (F(1, 39) = 46.99, *p* < 0.001, η^2^p = 0.55), with no significant main effect of group or time × group interaction.

For BDI-II depressive symptoms, the fascia-focused intervention group showed a decrease from 27.48 ± 11.41 at baseline to 18.17 ± 12.36 after treatment, corresponding to a mean reduction of 9.30 ± 13.42 points (95% CI 3.50 to 15.11). In the conventional active control group, BDI-II scores decreased from 24.83 ± 11.59 to 17.61 ± 12.53, corresponding to a mean reduction of 7.22 ± 8.57 points (95% CI 2.96 to 11.48). The between-group difference in reduction was 2.08 BDI-II points (95% CI −4.91 to 9.07), again with the confidence interval including zero. Within-group effect sizes were moderate to large (Cohen’s dz = 0.69 for the fascia-focused group and dz = 0.84 for the control group). The between-group effect size for change was small (Cohen’s d = 0.18). Repeated-measures ANOVA confirmed a significant main effect of time for BDI-II scores (F(1, 39) = 20.65, *p* < 0.001, η^2^p = 0.346), with no significant main effect of group or time × group interaction.

Overall, these findings indicate statistically significant and clinically interpretable improvements in pain intensity and depressive symptom severity over time in both treatment groups, without evidence of between-group superiority for either rehabilitation package.

For the secondary and exploratory physical outcomes, pressure pain thresholds measured at thoracic and lumbar levels did not change significantly over time. Examination of spinal posture, mobility, and stability using the TricuroGO system revealed several time effects. Lumbar mobility increased significantly during the rehabilitation period, while the lumbar lordosis angle decreased. Thoracic mobility decreased significantly, accompanied by increased stiffness of thoracic tissue. Pelvic stability showed a modest but significant improvement over time. No meaningful alterations were observed in CVA or pelvic mobility. Importantly, none of these physical outcomes showed significant group effects or time × group interactions, indicating that observed changes were not specific to either treatment group. Repeated-measures ANOVA outcomes are presented in Table 3.

Treatment perception and acceptance, treatment credibility/expectancy, and pain-related behavior were collected as additional patient-reported variables to characterize treatment-related aspects of the sample. Measures of FSV, HAQ, and CEQ remained stable, with no significant changes over time or between groups. These findings indicate that improvements in pain and function were consistently observed across participants, regardless of differences in therapeutic alliance, treatment expectancy, or pain-related behavior.

### 3.2. Treatment Completion

All 41 participants included in the final analysis completed the allocated rehabilitation program. The planned intervention schedule consisted of five Bowen therapy or progressive muscle relaxation sessions and ten circuit-training sessions over the five- to six-week inpatient rehabilitation period. These scheduled treatment sessions were delivered as part of the routine rehabilitation program. No participant included in the final analysis discontinued the allocated intervention.

## 4. Discussion

This study examined the effects of fascia-focused versus conventional physiotherapy on pain intensity, depressive symptom severity, and spinal function in psychosomatic inpatients with CLBP and depression. Both treatments were associated with significant improvements over time in pain intensity, depressive symptom severity, and selected postural and mobility measures, with no significant between-group differences or time × group interactions. The main finding is therefore that the fascia-focused rehabilitation package did not outperform the conventional active rehabilitation package on the measured outcomes.

This null between-group finding is clinically important because both interventions were active, structured, therapist-supervised, and embedded in the same inpatient psychosomatic rehabilitation environment. Therefore, the absence of superiority should not be interpreted as evidence that fascia-focused treatment is ineffective, but rather as an indication that its observed effects were not greater than those of an established active comparator in this exploratory sample. The parallel improvements in both groups may be explained by shared therapeutic mechanisms, including structured movement, physical activation, therapist attention, group-based care, treatment expectation, body awareness, and the broader multimodal rehabilitation context. Thus, the findings suggest that structured active rehabilitation and the overall therapeutic environment were likely important contributors to improvement, whereas the fascia-focused elements did not add measurable superiority over the conventional active program. Treatment selection may therefore reasonably depend on patient preference, therapist expertise, feasibility, and local rehabilitation resources.

To our knowledge, this is one of the first controlled clinical studies to directly compare a fascia-focused rehabilitation package with a conventional active physiotherapy and relaxation-based package in this specific psychosomatic population. The present findings should be interpreted in relation to the type of comparator and population studied in prior research. Previous studies of isolated myofascial or manual interventions have reported short-term improvements in pain-related outcomes or myofascial tissue properties, particularly when outcomes were assessed immediately after treatment [12,14]. However, such studies differ from the present study in that they primarily evaluated specific manual or fascia-directed interventions rather than multimodal rehabilitation packages embedded within a broader inpatient program. Similarly, rehabilitation-focused fascia approaches in CLBP populations have shown improvements in mobility, movement-related pain, and quality of life over several weeks [11]. However, improvements over time do not necessarily indicate superiority when the comparator is another active exercise-based or rehabilitation approach.

The present study extends this comparative question to psychosomatic inpatients with CLBP and comorbid depression, a population in which pain-related, affective, and functional impairments may interact [1,4,5]. In this context, both groups received active, structured, multimodal interventions within the same inpatient psychosomatic rehabilitation environment. The absence of superiority of the fascia-focused package, therefore, suggests that the observed improvements were not specific to fascia-focused treatment elements. Rather, shared therapeutic mechanisms, including guided movement, relaxation, therapeutic attention, structured care, body awareness, physical activation, and the broader rehabilitation context, may have contributed substantially to the improvements observed in both groups. Thus, the findings do not contradict previous reports of beneficial effects of fascia-oriented interventions, but they suggest that such effects may not translate into superior outcomes compared with an active, clinically plausible rehabilitation package in a complex psychosomatic population.

The parallel reductions in pain intensity and depressive symptoms support the clinical interpretability of the observed improvements in this psychosomatic inpatient population, in whom sensory, affective, and functional impairments coexist. This is in line with previous evidence indicating that multimodal or multidisciplinary treatment strategies that include physical activity can improve pain-related and psychosocial outcomes in CLBP populations, and it is also compatible with current recommendations emphasizing non-surgical, biopsychosocial approaches to CLBP management [6,32,33]. More broadly, recent evidence suggests that many non-surgical interventions for CLBP yield modest rather than large effects, which makes the absence of clear superiority between two active rehabilitation strategies plausible in an exploratory sample of this size [34]. The lack of a significant between-group difference may indicate that the observed benefits were driven less by fascia-specific effects alone and more by shared therapeutic mechanisms, such as guided movement, increased body awareness, activation, therapeutic attention, and the general benefits of participating in a structured rehabilitation program. This interpretation is consistent with previous trials showing that different active rehabilitation strategies can lead to similar improvements despite differing theoretical rationales [35]. It is also consistent with the view that in complex clinical populations with both CLBP and depression, treatment effects are likely shaped by multiple interacting physical and psychosocial pathways rather than by one isolated mechanism.

The clinical relevance of these findings should be interpreted cautiously because clinically meaningful change was not predefined and no responder analysis was performed. For pain intensity, reductions of approximately 2 points on an 11-point NRS or about 30% from baseline have been proposed as clinically important improvements in chronic pain populations [36]. In the present study, NRS and BDI-II scores decreased significantly over time in both groups. However, without predefined MCID thresholds, individual responder analyses, or assessment of BDI-II severity-category shifts, these findings should be interpreted as statistically significant and potentially clinically relevant improvements, but not as confirmed clinically meaningful improvement or remission at the individual level.

Although participants reported significant reductions in pain intensity, these subjective improvements were not mirrored by changes in PPT, which remained stable in both groups. This pattern is not necessarily contradictory, as PPT reflects experimentally evoked mechanical pain sensitivity, whereas the numeric rating scale captures the subjective experience of clinical pain. The two measures, therefore, assess related but distinct aspects of pain and may respond differently to rehabilitation interventions, particularly in patients with longstanding chronic pain [37]. A similar null result was observed for the CVA. This contrasts with the systematic review, by which found evidence that therapeutic exercise can improve forward head posture. However, those studies often used more specific corrective exercise programs and varied considerably in duration, whereas the present interventions were part of a broader multimodal rehabilitation approach and were not primarily designed as targeted treatments for cervical posture [38]. The lack of CVA change in this study should not be interpreted as evidence that postural variables are generally unresponsive, but rather that this specific rehabilitation format may not have been sufficiently targeted or long enough to produce measurable effects on this parameter.

Regarding spinal function, the current findings indicate improvements in lumbar and thoracic mobility as well as some postural outcomes, but they do not demonstrate a clear advantage of the fascia-focused approach over the conventional program. This is important clinically because it suggests that meaningful functional gains can be achieved through various active rehabilitation strategies. However, caution is warranted when interpreting changes in regional movement. Spinal segments do not function independently, and adaptive interactions between thoracic and lumbar regions are common during trunk movement. In this context, the idea of regional interdependence provides a reasonable explanation for the observed pattern of findings, although the data do not allow for definitive mechanistic conclusions [39].

An unexpected finding was the increase in thoracic tissue stiffness after rehabilitation. This contrasts with earlier studies that reported immediate reductions in fascial stiffness right after isolated myofascial interventions [12,14]. Several explanations are possible. First, the current study measured stiffness after a multi-session rehabilitation program instead of immediately following a single targeted intervention. Second, the interventions combined manual and active components, which may produce different tissue responses than isolated passive treatments. Third, the clinical significance of increased thoracic stiffness in this context remains unclear. It might indicate adaptive changes in motor behavior, posture, or load distribution, but could also be influenced by measurement variability, postural factors during assessment, or the uncertain clinical relevance of regional stiffness changes in a complex rehabilitation setting. Therefore, this result should be interpreted with caution and not regarded as definitive evidence of beneficial remodeling.

Several methodological limitations should be considered when interpreting the present findings. The study did not include an a priori sample size calculation, and the final sample size was relatively small for a study with multiple outcomes. Consequently, the analyses should be viewed as exploratory, and the absence of statistically significant between-group differences should not be interpreted as evidence that such differences do not exist, because small or moderate effects may have remained undetected.

Additionally, repeated-measures ANOVA was used in a two-time-point design, but this method does not adjust for potential baseline differences as directly as ANCOVA or linear mixed-effects modeling. This is relevant because the allocation procedure was quasi-randomized. Although baseline NRS and BDI-II depressive scores did not differ significantly between groups, residual baseline imbalance in other clinical or exploratory physical outcomes cannot be ruled out. Future studies should preferably use analytical approaches that adjust for baseline values, such as ANCOVA or linear mixed-effects models.

Because the study included multiple outcome measures and no formal correction for multiple comparisons was applied, the risk of type I error should be considered when interpreting isolated significant findings, particularly for posture-, mobility-, and stiffness-related outcomes. These findings should therefore be regarded as exploratory rather than confirmatory.

The interpretation of clinically meaningful change is also limited because predefined minimal clinically important difference thresholds, reliable change indices, or responder analyses were not applied. Therefore, clinical relevance can only be discussed descriptively, based on the direction of change, statistical significance, and effect size. This applies particularly to pain intensity and depressive symptom severity, whereas posture-, mobility-, and stiffness-related findings should be interpreted more cautiously as exploratory outcomes.

Baseline comparability was demonstrated for the available demographic variables as well as for baseline NRS and BDI-II. However, the clinical characterization of the sample remained limited. Pain duration, pain subtype, pain distribution, medication use, relevant somatic or psychiatric comorbidities, prior treatment history, adherence, and additional components of the routine inpatient rehabilitation program were not systematically documented or controlled analytically. Therefore, residual confounding and clinical heterogeneity cannot be ruled out, particularly given the quasi-randomized allocation procedure.

Although no between-group differences were observed, a possible influence of group setting and social context cannot be fully excluded. Furthermore, no intention-to-treat analysis was conducted, and the results are based on complete cases after excluding incomplete datasets and cases of early termination of rehabilitation. Session-level adherence and compliance rates, exact exercise intensity, completed circuit rounds, progression decisions, individual adaptations, and systematic adverse event data were not available in the dataset. Although the overall circuit structure, timing, number of stations, and graded exercise options were standardized, the practical selection and adaptation of exercise difficulty were left to the supervising physiotherapist. Additional limitations relate to the measurement procedures. Outcomes were assessed only at baseline and after the rehabilitation period; immediate post-session and long-term follow-up data were not collected. Assessor blinding was not practical in the clinical setting.

In addition, the study compared two multimodal rehabilitation packages rather than isolated treatment components. The fascia-focused rehabilitation package combined Bowen therapy with fascia circuit training, whereas the conventional active rehabilitation package combined progressive muscle relaxation with strength circuit training. Because multiple elements differed between groups, including the manual or relaxation component and the content of the circuit training, the study does not allow conclusions about which specific component was responsible for the observed effects or whether any changes can be attributed specifically to fascia-focused treatment elements.

Despite these limitations, the study has important clinical implications. Both treatment methods led to improvements in pain, depressive symptoms, and specific functional measures, indicating that both can be incorporated into psychosomatic inpatient rehabilitation as active treatment options. Since no clear evidence showed that the fascia-focused program was superior, treatment choices in clinical practice can reasonably be based on patient preference, therapist expertise, practicality, and the local rehabilitation setting rather than assuming one approach is generally more effective. Given the quasi-randomized design, the modest sample size, and the exploratory character of the study, these findings should be interpreted as indicating similar observed effects in this sample rather than formal equivalence between the two approaches.

Future studies should use fully randomized designs, larger sample sizes, and longer follow-up periods, and include more detailed documentation of confounders, adherence, and adverse events. It would also be helpful to investigate whether specific patient subgroups derive greater benefit from fascia-focused approaches and whether changes in tissue stiffness, motor control, or autonomic regulation influence treatment response. Such research would help determine if fascia-focused interventions provide additional value beyond traditional active rehabilitation strategies.

## 5. Conclusions

In this exploratory quasi-randomized study of psychosomatic inpatients with CLBP and comorbid depression, fascia-focused and conventional physiotherapy showed similar observed effects on the measured outcomes. Both treatments were associated with improvements over time in pain intensity, depressive symptom severity, and several postural and mobility measures. However, the study did not demonstrate superiority of one intervention over the other and was not designed or powered to establish formal equivalence. Because both interventions were delivered as multimodal treatment packages, the findings do not allow conclusions about which specific component was responsible for the observed improvements. Since the study did not assess a combined treatment approach, the current findings also do not support conclusions about their combined or additive benefits.

## Figures and Tables

**Table 1:** Baseline demographic characteristics.

Variable	Intervention Group (*n* = 23)	Control Group (*n* = 18)	*p*-Value
female/male (*n*, %)	15(65.2)/8(34.8)	12(66.7)/6(33.3)	0.92
Age (years)	52 ± 9.9	53.4 ± 6.2	0.78
Height (cm)	169.8 ± 8.8	173.8 ± 10.7	0.45
Weight (kg)	81.1 ± 14.1	80.1 ± 14.1	0.96
BMI (kg/m^2^)	28.5 ± 5.8	26.3 ± 3.9	0.52
NRS	6.99 ± 1.90	6.49 ± 1.92	0.26
BDI-II	27.48 ± 11.41	24.83 ± 11.59	0.53

Data are presented as mean ± standard deviation or *n* (%). BMI: Body Mass Index; SD: Standard Deviation; NRS: Numeric Rating Scale; BDI-II: Beck Depression Inventory-II.

**Table 2:** Clinical change in NRS pain intensity and BDI-II depressive symptom severity.

Outcome	Group/Comparison	Baseline Mean ± SD	Post-Treatment Mean ± SD	Absolute Reduction Mean ± SD (95% CI)	Effect Size
**NRS**	Fascia-focused intervention (*n* = 23)	6.99 ± 1.90	3.78 ± 2.58	3.21 ± 2.61 (2.08 to 4.34)	1.23
	Conventional active control (*n* = 18)	6.49 ± 1.92	4.32 ± 3.09	2.17 ± 2.34 (1.01 to 3.33)	0.93
	Between-group difference in reduction	-	-	1.04 (−0.53 to 2.60)	0.42
**BDI-II**	Fascia-focused intervention (*n* = 23)	27.48 ± 11.41	18.17 ± 12.36	9.30 ± 13.42 (3.50 to 15.11)	0.69
	Conventional active control (*n* = 18)	24.83 ± 11.59	17.61 ± 12.53	7.22 ± 8.57 (2.96 to 11.48)	0.84
	Between-group difference in reduction	-	-	2.08 (−4.91 to 9.07)	0.18

Data are presented as mean ± standard deviation unless otherwise indicated. Absolute reduction was calculated as the baseline value minus the post-treatment value; therefore, positive values indicate improvement. The 95% confidence intervals refer to the mean reduction within each group or to the between-group difference in reduction. Within-group effect sizes are reported as Cohen’s dz based on individual change scores; between-group effect sizes are reported as Cohen’s d for change scores. BDI-II: Beck Depression Inventory-II; CI: confidence interval; NRS: numeric rating scale; SD: standard deviation.

**Table 3:** Repeated-measures ANOVA results.

Outcome	Time			Group			Time × Group		
	F (1, 39)	*p*	η^2^p	F (1, 39)	*p*	η^2^p	F (1, 39)	*p*	η^2^p
NRS	46.99	<0.001	0.550	0.002	0.970	0.000	1.74	0.190	0.040
BDI-II	20.645	<0.001	0.346	0.236	0.630	0.006	0.328	0.570	0.008
Stiffness of lumbar fascia	3.61	0.070	0.090	0.07	0.790	0.002	1.46	0.230	0.040
Stiffness of thoracic fascia	9.99	0.003	0.200	0.41	0.530	0.010	0.02	0.890	0.000
Lumbar PPT	0.001	0.970	0.000	0.001	0.980	0.000	0.01	0.920	0.000
Thoracic PPT	0.74	0.400	0.020	0.07	0.800	0.002	0.20	0.660	0.005
Thoracic posture	3.27	0.080	0.080	1.13	0.290	0.030	0.01	0.940	0.000
Lumbar posture	9.67	0.003	0.200	1.10	0.300	0.030	0.06	0.810	0.002
Pelvic posture	21.81	0.001	0.350	0.58	0.450	0.020	0.08	0.780	0.080
Thoracic mobility	20.69	0.001	0.350	2.70	0.120	0.070	0.01	0.940	0.000
Lumbar mobility	28.95	0.001	0.430	1.37	0.250	0.030	0.28	0.600	0.010
Pelvic mobility	0.64	0.430	0.020	3.68	0.060	0.090	0.43	0.520	0.010
Thoracic stability	0.38	0.540	0.010	1.84	0.180	0.050	0.03	0.860	0.001
Lumbar stability	1.34	0.250	0.030	0.70	0.410	0.020	0.08	0.780	0.010
Pelvic stability	4.91	0.030	0.110	0.33	0.570	0.010	0.001	0.980	0.000
CVA	1.59	0.210	0.020	1.61	0.210	0.040	0.96	0.330	0.020

Main effects of time and group and time × group interactions are shown for clinical and exploratory physical outcomes. BDI-II: Beck Depression Inventory-II; CVA: craniovertebral angle; NRS: numeric rating scale; PPT: pressure pain threshold; ηp^2^: partial eta squared.

## Data Availability

The datasets generated and analyzed during the current study are available from the corresponding author on reasonable request.

## Supplementary Materials

The following supporting information can be downloaded at: https://www.mdpi.com/article/10.3390/jcm15103698/s1. Table S1. Overview of the exercises included in the strength circuit and fascia circuit-training programs. The table summarizes the six exercise stations used in each circuit-training program. Exercise titles were reconstructed from the original training materials. The brief descriptions and target functions are intended to improve reproducibility and provide an overview of the exercise content; they should therefore be interpreted as structured summaries of the intervention rather than as full standardized exercise protocols. Both circuit formats were conducted with six stations, each performed for 1 min, followed by a 1 min transition period. Depending on the available session time, approximately 1.5 to 2 rounds were completed per session.
