# Supplementary material for: Fascia-Focused Versus Conventional Physiotherapy for Chronic Low Back Pain and Comorbid Depression in Psychosomatic Inpatients
Source: J Clin Med. 2026 May 11;15(10):3698. doi: 10.3390/jcm15103698 (PMC13207669; doi:10.3390/jcm15103698)
Supplement: Supplementary file 1 [file jcm-15-03698-s001.zip › jcm-4285052-supplementary.pdf]

Supplementary Material

Supplementary Table S1. Overview of the exercises included in the strength circuit and fascia circuit training programs.

The table summarizes the six exercise stations used in each circuit-training program. Exercise titles were reconstructed from the original training materials. The brief descriptions and target functions are intended to improve reproducibility and provide an overview of the exercise content; they should therefore be interpreted as structured summaries of the intervention rather than as full standardized exercise protocols. Both circuit formats were conducted with six stations, each performed for 1 minute, followed by a 1-minute transition period. Depending on the available session time, approximately 1.5 to 2 rounds were completed per session.

Group	Station	Exercise name	Brief exercise description	Likely target / purpose
Strength circuit	1	Supine bridge	Bridge exercise performed in the supine position.	Trunk and hip extensor strength; lumbopelvic stability
Strength circuit	2	Side plank	Side-support exercise performed in a lateral position.	Lateral trunk stability; oblique and hip stabilizer activation
Strength circuit	3	Chair exercise	Exercise performed using a chair as support or reference.	Functional lower-limb and trunk control
Strength circuit	4	Prone exercise	Exercise performed in the prone position.	Posterior chain and trunk extensor activation
Strength circuit	5	Supine abdominal exercise	Abdominal exercise performed in the supine position.	Trunk flexor strength; abdominal control
Strength circuit	6	Quadruped position	Exercise performed in a four-point kneeling position.	Core stability; spinal control; coordinated limb-trunk activation
Fascia circuit	1	Dumbbell swinging	Swinging movement using a dumbbell.	Dynamic myofascial loading; elastic recoil; whole-body coordination
Fascia circuit	2	Cherry pit bag throwing	Throwing movement using a small beanbag/cherry pit bag.	Dynamic coordination; rotational and upper-body myofascial activation
Fascia circuit	3	Foam rolling	Self-myofascial rolling exercise using a fascia roller.	Myofascial stimulation; tissue gliding; mobility
Fascia circuit	4	Jumping	Rebound or jumping exercise.	Elastic loading; spring-like lower-limb and fascial function
Fascia circuit	5	Redondo ball exercise	Exercise performed with a Redondo ball.	Trunk control; dynamic stability; elastic resistance
Fascia circuit	6	Leg swing	Repetitive swinging movement of the leg.	Dynamic mobility; myofascial chain activation; coordination
